# Biosynthesis of Poly-ß-Hydroxybutyrate (PHB) from Different Bacterial Strains Grown on Alternative Cheap Carbon Sources

**DOI:** 10.3390/polym13213801

**Published:** 2021-11-03

**Authors:** Sherif M. El-Kadi, Mohssen Elbagory, Hassan A. H. EL-Zawawy, Hossam F. A. EL-Shaer, Adel A. Shoukry, Sahar El-Nahrawy, Alaa El-Dein Omara, Dina Fathi Ismail Ali

**Affiliations:** 1 Agricultural Microbiology Department, Faculty of Agriculture, Damietta University, Damietta 22052, Egypt; sherifelkadi@du.edu.eg; 2 Department of Biology, Faculty of Science and Arts, King Khalid University, Mohail Assir 61321, Saudi Arabia; mhmohammad@kku.edu.sa; 3 Department of Microbiology, Soils, Water and Environment Research Institute, Agricultural Research Center, Giza 12112, Egypt; sahar.elnahrawy@yahoo.com; 4 Botany Department (Microbiology), Faculty of Agriculture, Al-Azhar University, Cairo 11651, Egypt; zawawy.hassan@gmail.com (H.A.H.E.-Z.); alshokry17@azhar.edu.eg (A.A.S.); 5 Botany Department (Genetics), Faculty of Agriculture, Al-Azhar University, Cairo 11651, Egypt; hosamelshaer805@azhar.edu.eg; 6 Agricultural Microbiology Department, Faculty of Agriculture, Mansoura University, Mansoura 35516, Egypt; dfali@mans.edu.eg

**Keywords:** agro-industrial waste, GC-MS/MS spectrometry, IR spectrometry, PHB

## Abstract

Thirty bacterial isolates were tested on three different media for Poly-ß-hydroxybutyrate (PHB) production. The best bacterial isolates for producing PHB were screened and identified based on molecular biology; then, using three different alternative carbon sources (dried whey, sugar beet molasses and date molasses), physical properties were evaluated by Infrared (IR) spectrometry and Gas chromatography–mass spectrometry (GC-MS/MS) analysis. Our results showed that the best isolates identified based on molecular biology were *Bacillus paramycoides* MCCC 1A04098, *Azotobacter salinestris* NBRC 102611 and *Brevundimonas naejangsanensis* BIO-TAS2-2. The addition of sugar beet molasses to the medium of *A. salinestris* increased the cell dry weight (CDW), PHB concentration, PHB% and conversion coefficient (4.97 g/L, 1.56 g/L, 31.38% and 23.92%, respectively). The correlation coefficient values between PHB g/L and CDW g/L varied between very strong and moderate positive correlation. IR of the produced PHB from *B. paramycoides* and *A. salinestris* showed similar bands which confirmed the presence of PHB; however, *B. naejangsanensis* showed weak bands, indicating lower PHB concentration. The chemical composition obtained showed that the GC-MS of the PHB extracted represents 2, 4-ditert-butylphenol for *B. paramycoides* and isopropyl ester of 2-butenoic acid for both of *A. salinestris* and *Brevundimonas naejangsanensis*. Therefore, PHB produced by microorganisms can be considered a biodegradable polyester, and represents a promising technique for the development of eco-friendly and fully biodegradable plastics.

## 1. Introduction

Since petroleum-based plastics have become a major cause of environmental pollution, degradable plastics have attracted a great deal of attention worldwide for their unique properties, such as thermoplasticity. However, production of these vital plastics is limited due to their high cost of production. Hence, studies looking for bacterial strains with better yields and improving their culture conditions are of the greatest importance in order to reduce the costs of PHB production [1]. The slow growth of the PHB market since the 1980s is due to the high cost of production for the amount of PHB produced. Currently, only about 1% of plastic materials on the market are biodegradable, and with the increase in environmental pollution, there is a need to increase the production of PHB [2]. PHB and different biodegradable polyesters are promising tools for the development of eco-friendly and fully biodegradable plastics. PHBs are a class of biopolymers produced by different microbial species. Unlike plastics derived from petrochemicals, PHB is biodegradable and biocompatible in nature. PHBs have advanced applications in the medical sector (surgical pins and sutures, wound dressings, bone replacements), the packaging industry (packaging films, bags, cosmetics containers, shampoo bottles), and agriculture (plastic mulch, as a carrier for long-term doses of herbicides, fungicides and insecticides) [3,4,5,6]. PHB is produced using various raw materials such as agricultural and food industry wastes, which contributes to its economic feasibility. Research is currently concerned with reducing the cost of production [7]. The use of food industrial byproducts in the production of PHB is considered one of the growing areas of modern biotechnology; these products can be used as a nutrient-rich medium at no additional cost. Whey and molasses are very important byproducts that are rich in nutrients and which bacteria can convert into PHB [8]. The use of molasses as a potential low-cost substrate for PHB biopolymer production has been studied by [9]. The use of molasses, which is a cheap substrate available in Egypt, may help reduce the cost of producing such biopolyesters [3]. Beet molasses has been shown to be an excellent starting material for PHB production by *Azotobacter vinelandii*. The substrate cost for PHB production from beet molasses in fed-batch culture was one-third of that using glucose [10]. The results obtained by [11] indicate that by using *Bacillus subtilis* RS1, agro-industry wastes such as sugarcane molasses can be used as an inexpensive substrate for PHB production. Date molasses and date seed oil have also been used as the sole carbon source for PHB biosynthesis [12]. Processes are currently underway to improve PHB production via genetic modification of some strains in order to develop desirable and suitable properties for some diverse applications [7]. Pavan [13] aimed to conduct an economic assessment of PHB production process using molasses. The alternative sources showed equally low production costs (USD 4.28 per kg) and better economic indicators among the evaluated scenarios. In recent years, using renewable raw materials for PHB production as degradable bioplastics has been a good method of environment consideration. Low-cost carbon substrates have the potential to reduce PHB production cost and increase sustainability [14]. According to [11], the global PHB market is expected to reach USD 93.5 million by 2021. In the Middle East and Arab countries, Date palm (*Pheonix dactylifera* L.) is an important commercial crop. Egypt is one of the largest date producers among Arab and world countries. Egyptian date production in 2020 was 1.9 million tons, which represented 21.2% of world production. Date soft types are highly perishable and easily attacked by microbial enzymes; thus, date and date molasses are considered good materials for fermentation processes [15,16,17]. The main reason for the low prevalence of bioplastic products is the high cost of production.

Therefore, this study aims to produce PHB using efficient PHB-producing bacteria on different available alternative carbon sources (whey, sugar beet molasses and date molasses) and to study their properties by infrared (IR) spectroscopy and gas chromatography GC-MS/MS mass spectrometry analysis.

## 2. Materials and Methods

### 2.1. Bacterial Isolation

Four different soil samples were obtained from Damietta and Dakahlia Governorates (New Damietta city, Kafer El-Batekh, Kafer Saad, and Kalabshou), Egypt, were used for bacterial isolation. From these soil samples, 30 bacterial isolates were randomly isolated on three different selective media (10 for each medium): medium (A) [18], medium (B) [19] and medium (C) [20], for PHB production. These isolates were identified in the Agricultural Microbiology Department, Faculty of Agriculture, Damietta University, Damietta, Egypt. Morphological and physiological characteristics of bacterial isolates such as shape, Gram stain, spore stain, capsule stain and motility were microscopically studied, as were indole and catalase tests, starch hydrolysis and casein hydrolysis [21,22,23].

Isolates obtained from each of the three previously-mentioned media (10 randomly picked from each medium) were maintained on slants of Nutrient agar medium [24], Modified Ashby’s medium [25], and Nutrient-rich medium [26], incubated at 5 °C, and sub-cultured monthly.

### 2.2. Efficacy of PHB-Producing Isolates

#### 2.2.1. Cultivation Media

A cultivation process was implemented so that isolates from each medium could be cultivated on the same isolation media for PHB production. Carbon and nitrogen sources for each medium were sterilized separately and were aseptically added before inoculation and after cooling of each medium.

For medium (A) [18], 20 g/L glucose (carbon source) and 2.0 g/L ammonium acetate (nitrogen source) were added to the sterilized medium. The pH was adjusted to 7. For to medium (B), 10 g/L fructose was added as a carbon source [19] and 1.2 g/L ammonium acetate (nitrogen source) was added to the sterilized medium; the pH was adjusted to 7.2. Finally, 30 g/L sucrose was used as a carbon source for medium (C) [20], and 1 g/L ammonium sulfate was added to the sterilized medium. The pH was adjusted to 6.8.

#### 2.2.2. Cultivation System

Batch cultivation on conical flasks was used in this experiment. Twenty ml of each medium was prepared from maintenance cultures to be used as an inoculum. Incubation was performed in an incubator shaker at 200 rpm at 30 °C for 48 h. The inoculum was aseptically transferred to 50 mL of the same medium and incubated under the same conditions for the same period mentioned above [27]. All experiments were conducted in three replications.

#### 2.2.3. Biomass Determination

A 50 mL sample of each culture was centrifuged at 5000× *g* for 10 min. Cell pellets were washed twice with sterile distilled water. The precipitated biomass was dried at 100 °C for 24 h or until a constant weight was reached. This value was used for determination of cell dry weight (CDW) [28].

### 2.3. PHB Determination

Ten ml of chloroform was added to the dried cell biomass obtained from the previous centrifuged cultures and incubated at 70 °C for 10 min, then centrifuged at 5000× *g* for 10 min. The resulting solution was separated and collected, and the chloroform was allowed to evaporate in order to obtain PHBs for further assessment. Then, the precipitate was dried at 100 °C for 24 h or until a constant weight was reached. This value was used for PHB–free cell dry weight determination [19]. The difference in weight between the previous two values was considered as PHB production (g/50 mL); this value was calculated by [29,30], as g/L:

PHB (%) = PHB weight (g/L) × 100/total cell dry weight (g/L)

Conversion coefficient (%) = PHB weight (g/L) × 100/utilized sugar weight (g/L)

### 2.4. Determination of Utilized Sugars and Nitrogen

Total carbohydrates were determined as glucose [31]. The total sugars remaining in the culture broth were estimated, and by subtracting this value from the amount of total sugars in the medium composition at the beginning of the experiment, the consumed sugars or utilized sugars were calculated. Total nitrogen was measured according to the Kjeldahl digestion method [32].

### 2.5. The Relationship between PHB Production, CDW and Utilized Sugar (g/L)

The correlation coefficient values (*r*) was calculated according the equation given in [33]. The correlation between PHB g/L and CDW g/L was calculated where *X* is the value of PHB g/L,$\;\bar{X}$ is the arithmetical mean of PHB g/L, *Y* is the value of CDW g/L, and $\bar{Y}$ is the arithmetical mean of the CDW g/L values. The value of CDW was replaced with the value of utilized sugar when the correlation coefficient was calculated between PHB g/L and utilized sugar.

$$r=\frac{\sum \left(X-\bar{X}\right)\left(Y-\bar{Y}\right)}{\sqrt{\sum {\left(X-\bar{X}\right)}^{2}}\sqrt{\sum {\left(Y-\bar{Y}\right)}^{2}}}$$

If the value of the correlation coefficient (*r*) = +1, there is a perfect positive correlation; if (*r*) = −1, a perfect negative correlation; if *r* = 0, there is no correlation. If the value of the correlation coefficient is between *r* = 0.0–0.2, 0.2–0.4, 0.4–0.6, 0.6–0.8 or 0.8–1.0, this means a very weak correlation, weak correlation, moderate correlation, strong correlation or very strong correlation, respectively [33].

### 2.6. Identification of Bacterial Isolates Based on Molecular Biology

The highest PHB-producing isolates were inoculated in suitable media and incubated overnight at 37 °C. The resulting bacterial suspension was pelleted at 10,000 rpm for 5 min and genomic DNA was extracted using a Pure Link genomic DNA mini kit. PCR amplification of 16s rDNA was performed using the isolated DNA. The PCR conditions were set as follows: initial denaturation at 95 °C for 5 min, followed by 25 cycles of denaturation at 95 °C for 40 s, annealing at 55 °C for 2 min and primer extension at 72 °C for 1 min, ending with the final elongation step at 72 °C for 7 min. PCR products were gel purified and sent for sequencing with 16S rRNA primer. The obtained sequences were trimmed to obtain a sequence with ladder 1–1.5 Kb DNA. Then, the sequences were BLAST search analyzed on the (www.ncbi.nlm.nih.gov, accessed on 1 September 2021) to identify the isolate [34].

### 2.7. PHB Production Using Cheap Alternative Carbon Sources

Three different alternative carbon sources were used; dried whey and date molasses were purchased from Damietta City market, Damietta City, Egypt, and sugar beet molasses was purchased from the Sugar Beet Factory Kalabsho, Belkas, Dakahlia Governorate, Egypt. Molasses was pretreated according to the method in [35]. Total sugars and total nitrogen of all sources were determined. The values of carbon and nitrogen were considered during preparation of the cultivation media which already contain these sources.

### 2.8. The Physical Properties of PHB Polymer

IR was used to confirm the formation of PHB using a MATTISON 5000 FTIR spectrometer at Mansoura University, Faculty of Pharmacy, Chemistry Department, Spectral Analysis Unit. A 50 mm mortar and pestle were used. Hydraulic presses were performed using a PIKE die press. A SPECAC press instrument was used for preparation of the KBr sample discs of investigated drugs, pressing under a 7 mm die at a pressure of 2 tons for 1 min. A SHIMADZU CORPORATION balance BL-220H (Kyoto, Japan) was utilized throughout the work. IR spectra of the PHB polymer were determined at 2 cm^−1^ spectral resolution by using a MATTSON 5000 FTIR spectrometer. An excitation wavelength at 3900 nm was provided and the laser power at the sample position was typically 500 nm. Spectra were obtained with a spectral resolution of 4 cm^−1^, and 1024 [36].

For GC-MS/MS analysis, the chemical composition was obtained after extraction and derivatized using BSTFA reagent. The derivatization product MTBSTFA-HB was analyzed on a Finnigan GCQ (Finnigan MAT, Austin, TX, USA), which is an ion trap MS with external ionization. A 30 m fused silica capillary column DB-5MS, I.D. 0.25 mm, film thickness 0.25 mm (J&W Scientific, Folsom, CA, USA) was used for separation. The samples were injected in splitless mode. The injection temperature was 250 °C, temperature of ion source 200 °C and transfer line 275 °C. Helium velocity was 40 cm s^−1^. The oven temperature, initially at 60 °C, was gradually increased (20 °C min^−1^) to 250 °C and held at this temperature for 15.5 min. The MS/MS detection consisted of a sequence of three steps: (i) isolation of the “parent” ion of the detected compounds (immediate rejection of other ions except the “parent” ion); (ii) fragmentation of the “parent” ion; and (iii) analysis of the fragmentation products. These steps were realized using GCQ MS/MS software, version 2.0. The MS/MS measurements were performed at a collision energy of 0.8 V for parent ions of *m*/*z* 275 (for the analyte) and *m*/*z* 331 (for the internal standard). The product spectra in the mass range of *m*/*z* 100–331 were scanned. The retention time of the analyte was 8.34 ± 0.04 min and of the internal standard 10.2 ± 0.04 min [37].

### 2.9. Statistical Analysis

Data obtained throughout this study were analyzed by computer-assisted one-way ANOVA, using the software package stat graphics version 5.0 (costat). Least significance differences (LSDs) were calculated at level of significance *p* < 0.05 [38].

## 3. Results

### 3.1. Morphological and Physiological Characteristics of the Bacterial Isolates

Thirty bacterial isolates (10 isolates for each medium) were randomly isolated on three different selective media, medium (A), medium (B), and medium (C). Some of morphological and physiological characteristics of bacterial isolates were obtained. In addition, 10 bacterial isolates which were isolated on medium (A) were bacilli, Gram positive, spore positive, capsule positive and negative, and motile, and the indole test, catalase test, starch hydrolysis and casein hydrolysis were positive (Table S1, Supplementary Materials). From these characteristics, all isolates seemed to be *Bacillus* spp. [21]. Finally, 10 bacterial isolates which were isolated on medium (B) were diplococcic, Gram negative, non-spore, capsule positive, and motile and non-motile, and the indole test, catalase test, starch hydrolysis and casein hydrolysis were positive (Table S2, Supplementary Materials). From these characteristics, all isolates seemed to be *Azotobacter* spp. [21].

Bacterial isolates which were isolated on medium (C) were bacilli, cocci and short rods. Gram stain, spore stain, and capsule were positive and negative. Motile and none motile, indole test, catalase test, starch hydrolysis and casein hydrolysis were positive (Table S3, Supplementary Materials).

### 3.2. Screening the Best PHB-Producing Bacterial Isolates

The results presented in Table 1 show that all tested isolates cultivated on medium (A) produced PHB. The CDW varied from 0.98 to 2.99 g/L. PHB production ranged from 0.10 to 0.62 g/L. PHB% ranged between 6.84 and 27.55%. Sugar utilization ranged from 14.32 g/L to 18.51 g/L. The values of the conversion coefficient ranged between 0.62% and 3.90%. Significant differences were observed between bacterial isolates in most cases. Generally, the best isolate which yielded the highest PHB production with reasonable sugar utilization was No. A6. Therefore, this isolate was chosen and used in the subsequent experiments. Isolates No. A1, A2 and A6 also produced more than 20% PHB.

In addition, all tested isolates grown on medium (B) also produced PHB (Table 1). The highest values of cell dry weight, PHB production, PHB%, sugar utilization and conversion coefficient were 4.00 g/L, 0.93 g/L, 28.51%, 20.93 g/L, and 6.17%, respectively. However, the lowest values of the above parameters were 2.56 g/L, 0.73 g/L, 19.50%, 15.07 g/L, and 2.86%, respectively. It was observed that there were significant differences between the bacterial isolates; thus, the best isolate with the highest value of PHB (0.93 g/L) was No. P3. This isolate was therefore chosen for identification based on molecular biology and used in the experiments on alternative carbon sources. PHB was produced from all isolates grown on medium (C) (Table 1). The CDW values varied from 0.79 to 3.1 g/L. PHB production ranged from 0.08 to 0.73 g/L, and PHB% ranged between 6.04 and 23.67%. Sugar utilization ranged from 6.09 g/L to 8.17. The values of the conversion coefficient ranged between 1.00% and 8.93%. Generally, the best isolate which yielded the highest PHB production (0.73 g/L) with reasonable sugar utilization was No. W8. It was noted that there were significant differences between the bacterial isolates. Therefore, this isolate was chosen and used in the subsequent experiments.

### 3.3. Identification of the Best Bacterial Isolates Based on Molecular Method

Isolates No. A6, P3 and W8 (which were the best isolates for PHB production in the previous experiments) were identified according to the Polymerase Chain Reaction (PCR) method by Sigma Company (Cairo, Egypt) [34]. Isolate No. A6 was identified by the 16S ribosomal RNA gene and the partial sequence Query1 GAGCTTGCTCTTATGAAGTTAGCGGCGGACGGGTGAGTAACACGTGGGTAACCTGCCCAT 60.

**Figure 1:**
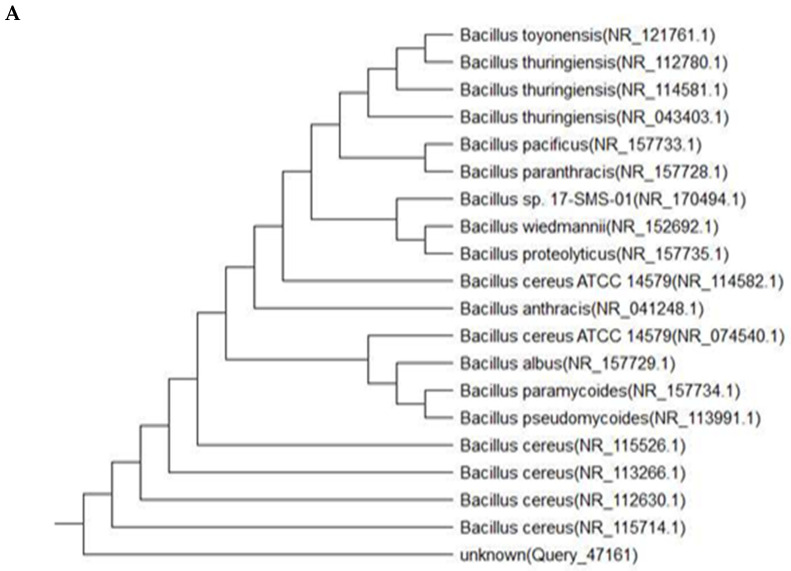

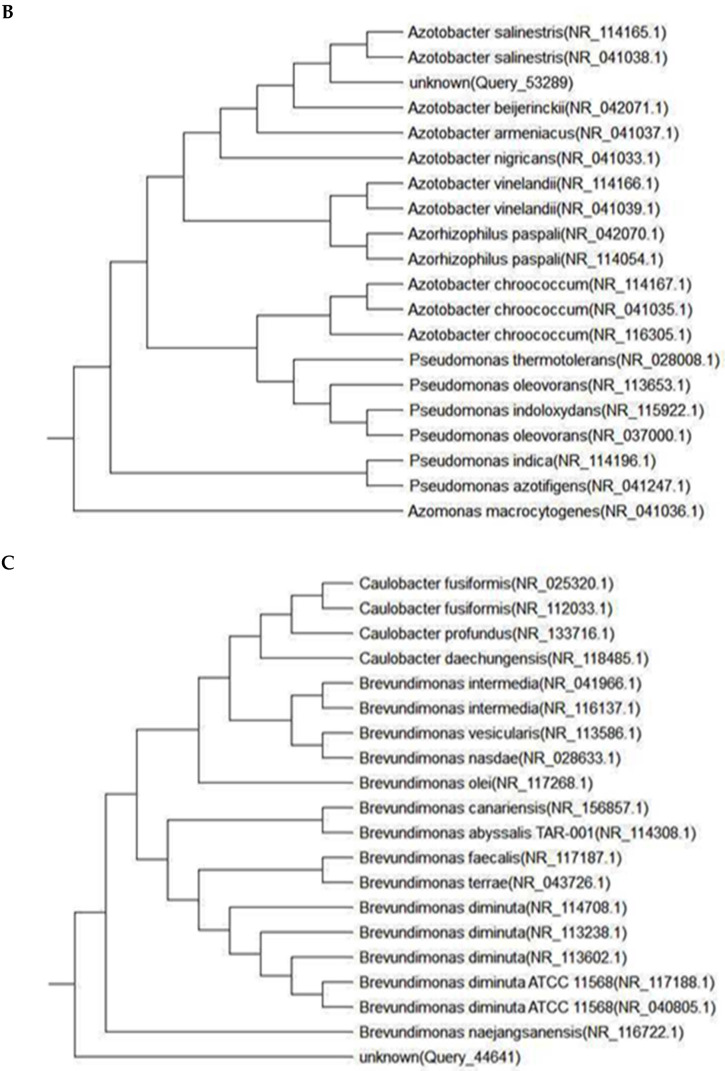
Genotype tree of *Bacillus paramycoides* MCCC 1A04098 (**A**), *Azotobacter salinestris* NBRC 102611 (**B**) and *Brevundimonas naejangsanensis* BIO-TAS2-2 (**C**).

The sequences of this isolate were accessed through a database (www.ncbi.nlm.nih.gov, accessed on 1 September 2021) using the accession number, and it was found to belong to the *Bacillus paramycoides* strain MCCC 1A04098 (Figure 1A). Isolate No. P3 was identified using the same method, by the partial sequence Query 1 AATACCCTGCAGTCTTGACGTTACCGGCAGAATAAGCACCGGCTAACTTCGTGCCAGCAG 60. It was identified as the *Azotobacter salinestris* strain NBRC 102611 (Figure 1B). Isolate No. W8 was also identified using the same method, by the partial sequence Query 1 GAATAACTCAGGGAAACTTGTGCTAATACCGAATGTGCCCTTTGGGGGAAAGATTTATCG 60. It was identified as *Brevundimonas naejangsanensis* strain BIO-TAS2-2 (Figure 1C).

### 3.4. PHB Production from Cheap Alternative Carbon Sources

The values of carbon and nitrogen were considered during preparation of the cultivation media, which already contained these sources. In dried whey, sugar beet molasses and date molasses, the carbon contents were 70%, 51%, and 37%, respectively, and the nitrogen contents were 15%, 13%, and 14.1%, respectively. Table 2 shows the effect on PHB production from *B. paramycoides* strain MCCC 1A04098 of replacing glucose in medium (A) with the three cheap alternative carbon sources (dried whey, treated sugar beet molasses, and treated date molasses). The addition of treated sugar beet molasses was the most economically suitable, as it increased PHB production (0.98 g/L) and the conversion coefficient (6.5%). Furthermore, the utilized sugar (15.11 g/L) was moderate and slightly lower than the control value (15.88 g/L). On the other hand, dried whey yielded the lowest PHB production (0.51 g/L), even lower than the control. The values of treated date molasses showed the same pattern as treated sugar beet molasses, except for sugar utilization which was the highest among all carbon sources (16.40 g/L). It was observed that there were significant differences between bacterial strains. In the present study, the maximum production of PHB and CDW by *B. paramycoides* was 0.98 g/L and 2.84 g/L from sugar beet molasses, respectively.

Fructose was replaced with alternative carbon sources in medium (B) for PHB production using the *A. salinestris* strain NBRC 102611. Table 2 showed that the use of treated beet molasses yielded the highest PHB production (1.56 g/L) and highest conversion coefficient (23.92%), with moderate sugar utilization (6.52 g/L) in comparison with the control values. Dried whey yielded the lowest PHB production (0.66 g/L). It was observed that there were significant differences between the bacterial isolates.

Sucrose was replaced with alternative carbon sources in medium (C) for PHB production from the *Brevundimonas naejangsanensis* strain BIO-TAS2-2. Table 2 showed that the use of treated date molasses yielded the highest PHB production (1.5 g/L) and the highest conversion coefficient (9.35%), with moderate sugar utilization (16.03 g/L) in comparison with the control values. Dried whey yielded the lowest PHB production (0.64 g/L). It was observed that there were significant differences between the bacterial isolates.

### 3.5. The Correlation Coefficient Value between PHB Production, CDW and Utilized Sugar (g/L) of All Tested Isolates and Strains

The correlation coefficient was calculated according the equation in [33]. Statistical analysis indicated there was a very strong positive correlation (r = 0.987) between PHB g/L and CDW g/L in the *A. salinestris* strain and a strong positive correlation (r = 0.457) in the isolates grown on medium (B). A very weak negative correlation (r = −0.161) between PHB g/L and utilized sugar g/L in the isolates grown on medium (B) was observed, with the highest value between them being r = 0.897 (a very strong positive correlation) in the case of *B. naejangsanensis* (Table 3).

### 3.6. The Physical Properties of PHB Polymer

#### 3.6.1. Fourier Transform Infrared Spectroscopy (FTIR)

**Figure 2:**
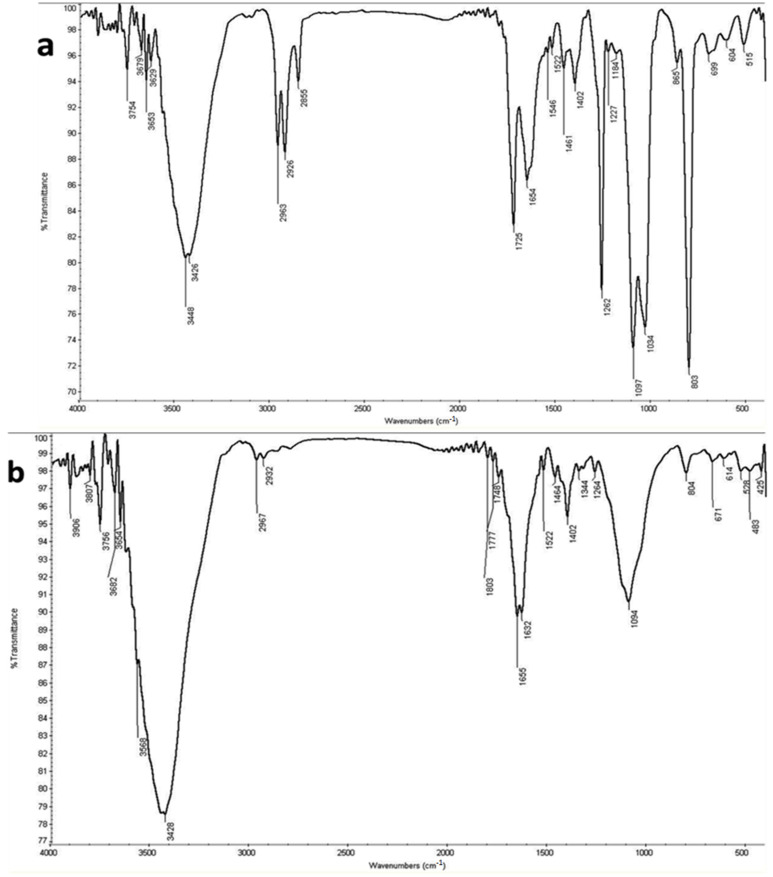

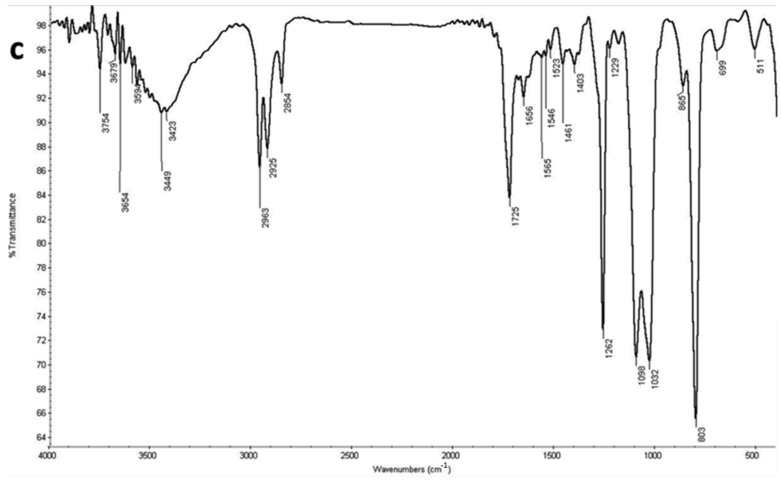
IR spectrum of PHB extracted from *B. paramycoides* (**a**), *A. salinestris* (**b**), and *Brevundimonas naejangsanensis* (**c**).

Poly ß-hydroxybutyrate (PHB) Polymer extracted from *B. paramycoides* was characterized using IR spectra in the range of 600–4000 cm^−1^, as displayed in Figure 2a. The IR spectra of PHB produced show obvious peaks at 1262 and 1725 cm^−1^, corresponding to specific rotations around carbon atoms specific to –CH and C=O stretching of the ester group present in the molecular chain [36]. Strong peaks at 1034 and 1097 indicate the presence of C-O stretching. Additionally, the absorption bands at 2926 and 2963 cm^−1^ are due to C–H stretching vibrations of methyl and methylene groups, which confirms PHB formation. PHB extracted from *A. salinestris*, as shown in Figure 2b, is similar to that extracted from *B. paramycoides*. Furthermore, the IR spectra of the extract from *A. salinestris* display similar bands at 1032, 1098 1262, 1725, 2925 and 2963 cm^−1^ which confirm the presence of PHB, as shown in Figure 2b. On the other hand, the IR spectra of PHB extracted from *Brevundimonas naejangsanensis* illustrate weak bands at 1264 and 1748 cm^−1^, indicating lower PHB concentration in comparison with the other obtained extracts, as shown in Figure 2c. In addition, all peaks at 3400 to 3600 cm^−1^ correspond to specific amino groups.

#### 3.6.2. Gas Chromatography–Mass Spectrometry (GC-MS) Analysis

**Figure 3:**
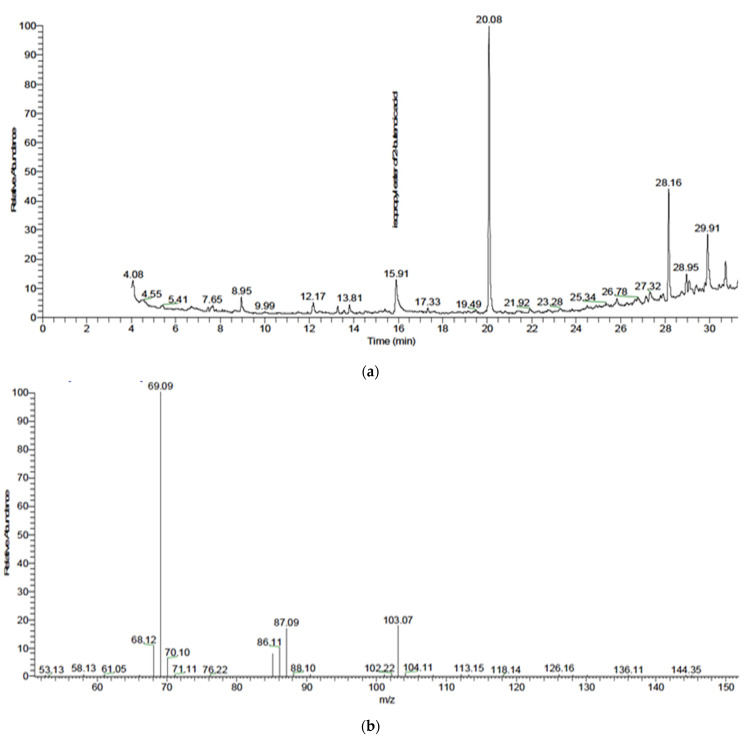
(**a**) Total GC-MS chromatogram of PHB extracted from *Bacillus paramycoides.* (**b**): The mass spectrum of isopropyl ester of 2-butenoic acid of PHB extracted from *Bacillus paramycoides*.

Chemical composition was obtained after extraction and derivatized using BSTFA reagent. Figure 3a shows a peak at retention time 15.91 min, which represents Polyhydroxybutyrate (PHB) from *B. paramycoides*. The molecular ion peak in the mass spectrum at *m*/*z* 104 agrees well with the molecular formula for PHB. In addition, Figure 3b shows a peak at 20.08 min, which represents 2, 4-ditert-butylphenol. The area under the peak denotes the PHB content, which was determined to be 4.85%.

**Figure 4:**
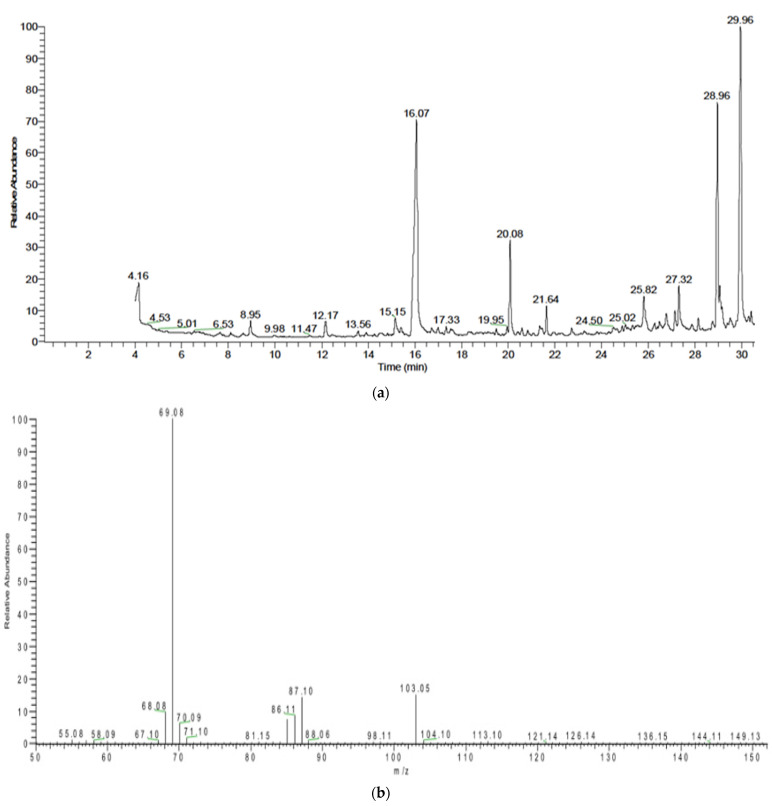
(**a**): Total GC-MS chromatogram of PHB extracted from *Azotobacter salinestris*. (**b**): The mass spectrum of isopropyl ester of 2-butenoic acid of PHB extracted from *Azotobacter salinestris*.

**Figure 5:**
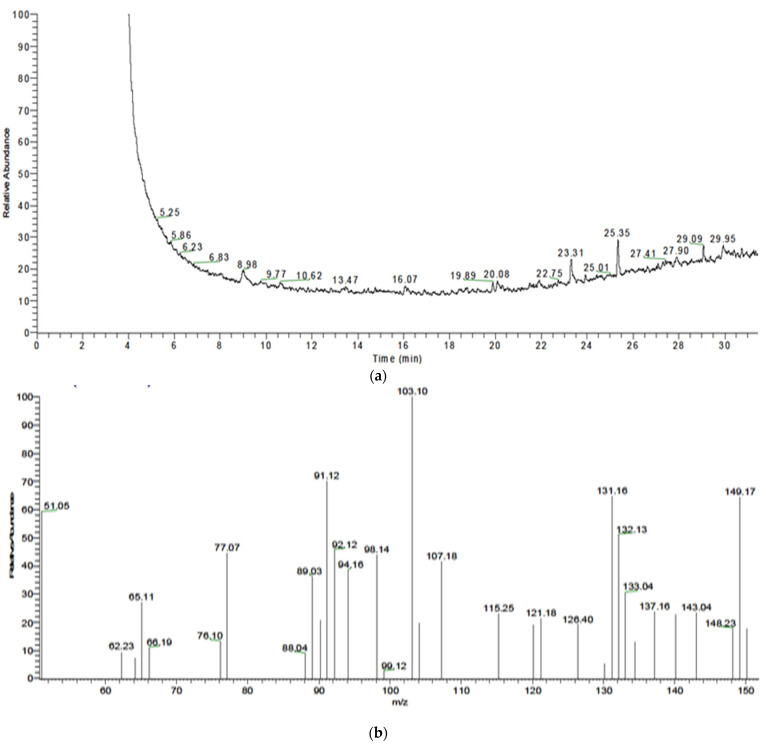
(**a**): Total GC-MS chromatogram of PHB extracted from *Brevundimonas naejangsanensis.* (**b**): The mass spectrum of isopropyl ester of 2-butenoic acid of PHB extracted from *Brevundimonas naejangsanensis*.

Figure 4a shows the GC-MS chromatogram of the PHB extracted from *A. salinestris*; the peak at retention time 16.07 min represents the isopropyl ester of 2-butenoic acid (Figure 4b), confirming the present of Polyhydroxybutyrate (PHB). The area under the peak denotes the PHB content, which was determined to be 10.73% (Figure 4b). Figure 5a shows the GC-MS chromatogram of the PHB extracted from *Brevundimonas naejangsanensis*, showing a peak at retention time 16.07 min which represents the isopropyl ester of 2-butenoic acid (Figure 5b), confirming the presence of Polyhydroxybutyrate (PHB). The area under the peak denoting the PHB content was determined to be 0.85%, with very low mass spectrum matching.

## 4. Discussion

### 4.1. Screening the Best PHB-Producing Bacterial Isolates

Significant differences were observed between the bacterial isolates in most cases. Generally, the best isolate which yielded the highest PHB production with reasonable sugar utilization was No. A6 (Table 1). Isolates No. A1, A2 and A5 produced more than 20% PHB. These results were similar to the results obtained by [39], who isolated three bacteria from the roots of beans (*Vicia faba*) grown in Qalyubia Governorate, Egypt to produce PHB using the flask culture technique. These isolates collected more than 20% of PHB, and were identified as *Pseudomonas fluorescens* S48, *B. megaterium* 7A and *B. megaterium* UBF19. Four isolates (A1, A2, A6 and A10) were higher than those found by [18], who reported that the highest value of PHB in *B. megaterium* was 0.27 g/L. Furthermore, all of these isolates were higher than [40], who produced PHB by batch fermentation of *B. subtilis* from glucose and reached only 0.077 g/L.

In addition, all tested isolates grown on medium (B) produced PHB (Table 1). It was observed that there were significant differences between bacterial isolates. Thus, the best isolate with the highest value of PHB (0.93 g/L) was No. P3. Multiple previous studies [19,41] produced PHB from *A. chroococcum* in amounts ranging from 2.0 to 3.0 g/L, higher than in the current study; however, [10] produced only about 1.0 g/L from *A. vinelandii*, lower than this study. In [42], the production of PHB was studied using the shaking culture method with *A. chroococcum* resulting in a PHB% of 46.80%. On the other hand, PHB was produced from all isolates grown on medium (C) in the present study (Table 1).

### 4.2. Identification of the Best Bacterial Isolates Based on Molecular Method

Isolates No. A6, P3 and W8 were identified according to the Polymerase Chain Reaction (PCR) method. The sequence of the A6 isolate was accessed using the accession number and it was found to belong to the *Bacillus paramycoides* strain MCCC 1A04098 (Figure 1A). Although several reports have produced PHB from *Bacillus* sp., such as *B. amyloliquefaciens*, *B. aryabhattai*, *B. brevis*, *B. cereus*, *B. circulans*, *B. coagulans*, *B. firmus*, *B. laterosporus*, *B. licheniformis*, *B. macerans*, *B. megaterium*, *B. sphaericus*, *B. subtilis*, and *B. thuringiensis* [1,14,43], and a few researchers [44,45,46,47,48] have used *B. mycoides* for producing PHB, to the best of our knowledge only this study has produced PHB from *B. paramycoides*.

In addition, the isolate No. P3 was identified as *Azotobacter salinestris* strain NBRC 102611 (Figure 1B). Three species of the genus *Azotobacter* sp. have commonly appeared in the literature on the production of PHB, including *A. chroococcum* [19,28,41,42,49], *A. vinelandii* [50,51,52,53], and *A. beijerinckii* [54,55,56]; however, only one used *A. salinestris* for PHB production [57] before this study. Isolate No. W8 was identified as the *Brevundimonas naejangsanensis* strain BIO-TAS2-2 (Figure 1C). Only one study has used *Brevundimonas naejangsanensis* for PHB production [58] prior to the current study. This study is also the first to report the newly isolated bacterial strain designated as *Bacillus paramycoides* strain MCCC 1A04098 as potential source for PHB production.

### 4.3. PHB Production from Cheap Alternative Carbon Sources

Table 2 shows the effect of replacing glucose in medium (A) with three cheap alternative carbon sources, namely dried whey, treated sugar beet molasses, and treated date molasses, on PHB production from *B. paramycoides* strain MCCC 1A04098. Generally, in order to decrease the cost of PHB and increase the cell dry weight, PHB concentration, PHB%, and conversion coefficient, the addition of treated sugar beet molasses in the medium of *B. paramycoides* is recommended.

*Bacillus* sp. is capable of producing hydrolytic enzymes, which can degrade agro-industrial and other wastes that can be utilized as carbon sources for PHB production [43]. These results were similar to [46], where the highest PHB yield was observed in *B. mycoides* DFC1 (1.28 g/L) using wheat starch, and lower than [47], where a maximum dry cell weight of 4.35 g/L was obtained. In addition, these results were higher than those obtained in [59], where the maximum PHB% obtained by *B. subtilis* was 13.02% ± 1.67%. As for the attempts that have been made to reduce the cost of production, there have been many attempts, such as [1], who reported that PHB production from *B. aryabhattai* showed good polymer accumulation in basal medium with glycerol after 48 h of incubation and accumulated 1.79 g/L. Hence, glycerol may be a better option as it is cheaper than glucose and can be obtained as a byproduct from industries such as biodiesel production. In the same trend, the current study was better than [3], who studied the effect of different carbon sources on PHB production by *B. megaterium*. The highest production of PHB was observed with cane molasses as the sole carbon source (40.8, mg/L). The same results were obtained by [60], who reported that sugar beet molasses could be a suitable substrate for the production of PHB with *B. megaterium*. Cell dry weight was 16.7 g/L and PHB production was 0.6 g/L with batch cultivation. Sugarcane molasses was also used as a sole carbon source by [11,61,62], using *B. cereus* and *B. subtilis* to obtain a PHB% of 57.5%, 49.9% and 44.7%, respectively.

Fructose was replaced with alternative carbon sources in medium (B) for PHB production from *A. salinestris* strain NBRC 102611 (Table 2). In [52] it was found that there are more than of 300 bacterial species that can produce PHB, of which only a few have been used on a commercial scale. Molasses and whey have been reported to be excellent substrates for PHB production [35,57,63]. Similar results were obtained by [60], who reported that the use of sugar beet molasses in PHB production led to a two- to three-fold increase in biomass production compared to the control experiment. In [10] it was reported that the results obtained with beet molasses were the best compared with other sugars, especially unrefined sugar; in addition, it was proven that it is not only a source of carbon, but also contains some other nutrients that may interfere with the synthesis of PHB. Thus, to decrease the cost of PHB, the addition of sugar beet molasses in the medium of *A. salinestris* to increase cell dry weight, PHB concentration, PHB% and conversion coefficient appears very promising.

Sucrose was replaced with alternative carbon sources in medium (C) for PHB production from *Brevundimonas naejangsanensis* strain BIO-TAS2-2 (Table 2). Both the current study and [12] used date molasses as a novel carbon source for *B. naejangsanensis* and *Cupriavidus necator* for PHB production. The current findings were homogeneous with [58], who tested the ability of *B. vesicularis* to accumulate PHB and found that with acid-hydrolyzed wood (sawdust) as the substrate, PHB production was improved. In order to reduce the production cost by 50% and make it as economical as possible, more than 96% of sugars must be consumed, and the cells must contain more than 90% PHB with a yield not less than 64% of the DCW.

### 4.4. The Correlation Coefficient Value between PHB Production, CDW and Utilized Sugar (g/L) of All Tested Isolates and Strains

Multiple studies [64,65] have found a positive correlation between PHB production and CDW with r = 0.999, which was similar to our results where r = 0.987 (Table 3). The available studies on the correlation between PHB production and CDW were insufficient, with little information available; [64,65,66] published that there was a positive correlation between PHB and CDW. On the contrary, Aslim [67], reported there was a low correlation between CDW and the PHB contents of *Lactobacillus* cultures. Thus, there is a need for further study on this point. In this context, [68] reported that the correlation between PHB synthesis and nitrogen fixation was little discussed in purple non-sulfur bacteria species. In keeping with this trend, [65,69] studied the correlation between PHB production, pH, and viscosity, finding that it was decreased or that no correlation could be observed. However, there are no studies dealing with the correlation between PHB production and the amount of sugar consumed, which is the most important economic factor affecting polymer production.

### 4.5. The Physical Properties of PHB Polymer

#### 4.5.1. Fourier Transform Infrared Spectroscopy (FTIR)

Poly ß-hydroxybutyrate (PHB) Polymer extracted from the studied strains was characterized using IR spectra in the range of 600–4000 cm^−1^, as displayed in Figure 2. The results were obtained by [14], which observed that 1624–1724 cm^−1^ was associated with the C=O stretching of the ester carbonyl bond. The bond at 1529 cm^−1^ was characteristic of the stretching and deformation vibration of the C-H group, and those at 2960 and 3277 cm^−1^ were characteristic of the stretching and deformation vibrations of the terminal OH groups. The PHB polymer functional groups were confirmed as C=O groups by FT-IR spectroscopy. In addition, analysis of the spectrum of PHB was observed at 3400, 1639, 3018, 2978, 2842, 1216, 1044 and 669–765 δ, respectively, for the (-OH) broad peak, (C=C) double bond, (≡C-H) acetylenic bond, (-CH3) methyl group, (-S-H) thiol weak adsorption, (≡C-O-C≡) ether, sulfoxide and (F-Cl) haloalkanes [41]. The same results were obtained by [70], who showed that the bands of PHB between 3300 and 3700 cm^−1^ were ascribed to O-H groups in the phenolic and aliphatic structures. The band at 2923 cm^−1^ was assigned to C-H stretching in aromatic methoxyl groups, or in methyl and methylene groups of the side chain. The band at 1645 cm^−1^ was attributed to unconjugated C=O stretching. The bands centered at 1508 and 1593 cm^−1^ were associated with aromatic structural vibrations. The bands between 1400 and 1450 cm^−1^ originated from C-H deformation coupled with the vibration from the aromatic ring. The bands at 1217 and 1128 cm^−1^ indicated guaiacyl and syringyl groups, respectively. Moreover, Rathika [11], reported that, the broad transmittance peak at 3281 cm^−1^ could be ascribed to the stretching of O–H groups. Peaks observed at 2924 and 2852 cm^−1^ were associated with the C-H stretching of bonds of methyl (CH3) and methylene (CH2) groups. The intense absorption peak at 1722 cm^−1^ was the characteristic carbonyl (C=O) stretching of ester groups in the extracted PHAs.

The 1H NMR spectrum showed the expected resonances for PHB as demonstrated by the methine group (-CH-) between 5.22 and 5.28 ppm, a methylene group (-CH2-) between 2.45 and 2.62 ppm, and the methyl group (-CH3) between 1.26 and 1.28 ppm, as in standard PHB. Scans of 13C NMR showed peaks at 169.13, 67.62, 40.81 and 19.76 ppm, which represent the carbonyl carbon (-C-), ester (-O-CH-) group, methylene (-CH2-) and the methyl (-CH3) groups, as shown in the standard. These results confirm the material as a homopolymer of 3-hydroxybutyrates, i.e., poly-3-hydroxybutyrate [1].

#### 4.5.2. Gas Chromatography–Mass Spectrometry (GC-MS) Analysis

Our observation in Figure 3, Figure 4 and Figure 5 were in agreement with [11], who detected PHAs extracted from *B. subtilis* RS1 cultured and processed in a medium of sugarcane molasses using GC–MS and revealed significant peaks at retention times 20.9, 23.1, and 23.8 min, corresponding to methyl esters of pentadecanoic acid and hexadecanoic acid, respectively. Furthermore, the results revealed that hexadecanoic acid methyl ester is the predominant monomer of PHA produced from *B. subtilis* RS1. In addition, Ramya [14] reported that the bioplastics produced by *B*. *cereus* RBL6 were characterized by GC–MS. *B*. *cereus* RBL6 was reported to produce methyl-3-hydroxy hexadecanoic acid, which belongs to the monomer chains of the medium-chain-length PHA class.

## 5. Conclusions

Our study aimed to use cheap and available alternative carbon sources (whey, sugar beet molasses and date molasses) to produce PHB using *Bacillus paramycoides*, *Azotobacter salinestris* and *Brevundimonas naejangsanensis*. The results showed that the addition of sugar beet molasses in the medium of *A. salinestris* increased the cell dry weight (CDW), PHB concentration, PHB% and conversion coefficient. In addition, statistical analysis indicated that the correlation coefficient values between PHB g/L and CDW g/L varied between r = 0.987 and r = 0.457 (very strong positive correlation and moderate positive correlation); however, these values were r = −0.161 and r = 0.897 when the correlation was calculated between PHB g/L and utilized sugar g/L. In addition, the IR of the produced PHB from *B. paramycoides* and *A. salinestris* showed similar bands, which confirmed the presence of PHB; however, *B. naejangsanensis* showed weak bands. The chemical composition obtained by GC-MS of the PHB extract represented 2, 4-ditert-butylphenol for *B. paramycoides*, and isopropyl ester of 2-butenoic acid for both of *A. salinestris* and *Brevundimonas naejangsanensis*.

## Figures and Tables

**Table 1:** Screening of different bacterial isolates to produce PHB on different media.

Isolate Number	C.D.W (g/L)	PHB (g/L)	PHB (%)	Utilized Sugar (g/L)	Conversion Coefficient (%)
	**Medium A**				
**A1**	2.99 ^a^	0.61 ^b^	20.40 ^c^	17.03 ^c^	3.58 ^b^
**A2**	2.52 ^b^	0.51 ^b^	20.23 ^b^	15.36 ^d^	3.32 ^b^
**A3**	1.27 ^f^	0.11 ^de^	8.66 ^h^	16.00 ^d^	0.68 ^e^
**A4**	1.69 ^d^	0.15 ^de^	8.87 ^h^	14.32 ^e^	1.04 ^d^
**A5**	1.17 ^fg^	0.13 ^de^	11.11 ^g^	18.51 ^a^	0.70 ^e^
**A6**	2.25 ^c^	0.62 ^a^	27.55 ^a^	15.88 ^d^	3.90 ^a^
**A7**	0.98 ^h^	0.17 ^d^	17.34 ^e^	16.85 ^c^	1.00 ^d^
**A8**	1.46 ^e^	0.10 ^e^	6.84 ^i^	15.94 ^d^	0.62 ^e^
**A9**	1.03 ^gh^	0.12 ^de^	11.65 ^f^	17.77 ^b^	0.67 ^e^
**A10**	1.58 ^de^	0.29 ^c^	18.35 ^d^	15.80 ^d^	1.83 ^b^
**LSD 0.05**	0.145	0.042	0.404	0.486	0.140
	**Medium B**				
**P1**	3.61 ^b^	0.85 ^b^	23.54 ^c^	15.34 ^e^	5.54 ^b^
**P2**	3.93 ^a^	0.83 ^b^	21.11 ^d^	19.58 ^c^	4.23 ^de^
**P3**	3.47 ^bc^	0.93 ^a^	26.80 ^ab^	15.07 ^e^	6.17 ^a^
**P4**	3.30 ^c^	0.83 ^b^	25.14 ^bc^	15.19 ^e^	5.46 ^b^
**P5**	4.00 ^a^	0.78 ^bc^	19.50 ^d^	15.44 ^e^	5.05 ^c^
**P6**	3.62 ^b^	0.78 ^bc^	21.54 ^d^	17.32 ^d^	4.50 ^d^
**P7**	2.78 ^d^	0.74 ^c^	26.62 ^ab^	19.17 ^c^	3.86 ^e^
**P8**	2.56 ^e^	0.73 ^c^	28.51 ^a^	20.93 ^b^	3.48 ^f^
**P9**	3.88 ^a^	0.82 ^b^	21.12 ^d^	19.40 ^c^	4.22 ^de^
**P10**	3.40 ^bc^	0.84 ^b^	24.75 ^bc^	29.33 ^a^	2.86 ^g^
**LSD 0.05**	0.165	0.053	1.571	0.455	0.365
	Medium C				
**W1**	1.37 ^d^	0.10 ^ef^	6.6 ^d^	7.94 ^a^	1.25 ^ef^
**W2**	2.04 ^b^	0.28 ^b^	13.72 ^b^	6.82 ^e^	4.10 ^b^
**W3**	1.85 ^c^	0.17 ^d^	9.37 ^c^	7.71 ^b^	2.20 ^d^
**W4**	1.15 ^e^	0.17 ^d^	15.40 ^b^	7.39 ^c^	2.30 ^d^
**W5**	1.36 ^d^	0.21 ^c^	14.73 ^b^	7.10 ^d^	2.95 ^c^
**W6**	1.98 ^b^	0.16 ^d^	8.25 ^cd^	6.09 ^f^	2.62 ^cd^
**W7**	1.82 ^c^	0.13 ^de^	7.68 ^cd^	7.65 ^b^	1.69 ^e^
**W8**	3.10 ^a^	0.73 ^a^	23.67 ^a^	8.17 ^a^	8.93 ^a^
**W9**	0.79 ^f^	0.11 ^ef^	14.79 ^b^	7.44 ^c^	1.47 ^ef^
**W10**	1.33 ^d^	0.08 ^f^	6.04 ^d^	7.96 ^a^	1.00 ^f^
**LSD 0.05**	0.121	0.032	1.748	0.203	0.433

C.D.W: cell dry weight; PHB: Poly-ß-hydroxybutyrate; A1–10: symbol for unknown bacterial isolate grown on medium (A) [15]; P1–10: symbol for unknown bacterial isolate grown on medium (B) [16]; W1–10: symbol for unknown bacterial isolate grown on medium (C) [17]. Means followed by different letters indicate significant differences between treatments according to Duncan’s test (*p* < 0.05).

**Table 2:** Effect of cheap alternatives carbon sources on PHB production by different strains of *Bacillus paramycoides*, *Azotobacter salinestris* and *Brevundimonas naejangsanensis*.

Alternatives Carbon Sources	C.D.W (g/L)	PHB (g/L)	PHB (%)	Utilized Sugar (g/L)	Conversion Coefficient (%)
	***Bacillus paramycoides*** **strain MCCC 1A04098**				
**Dried whey**	1.56 ^d^	0.51 ^d^	33.81 ^a^	13.74 ^b^	3.71 ^c^
**sugar beet molasses**	2.84 ^a^	0.98 ^a^	34.50 ^a^	15.11 ^ab^	6.50 ^a^
**Date molasses**	2.67 ^b^	0.88 ^b^	32.86 ^a^	16.40 ^a^	5.36 ^b^
**(Control, glucose)**	(2.25 ^c^)	(0.62 ^c^)	(27.55 ^b^)	(15.88 ^a^)	(3.90 ^c^)
**LSD 0.05**	0.123	0.079	4.499	1.391	0.804
	***Azotobacter salinestris* strain NBRC 102611**				
**Dried whey**	2.47 ^d^	0.66 ^d^	26.51 ^bc^	5.15 ^d^	12.81 ^c^
**Sugar beet molasses**	4.97 ^a^	1.56 ^a^	31.39 ^a^	6.52 ^c^	23.92 ^a^
**Date molasses**	4.61 ^b^	1.36 ^b^	29.45 ^ab^	6.83 ^b^	19.91 ^b^
**(Control, fructose)**	(3.10 ^c^)	(0.73 ^c^)	(23.67 ^c^)	(8.17 ^a^)	(8.93 ^d^)
**LSD 0.05**	0.227	0.047	2.951	0.220	0.984
	***Brevundimonas naejangsanensis* strain BIO-TAS2-2**				
**Dried whey**	1.63 ^d^	0.64 ^d^	39.27 ^b^	11.59 ^d^	5.51 ^b^
**Sugar beet molasses**	3.57 ^a^	1.36 ^b^	40.36 ^b^	16.71 ^a^	9.73 ^a^
**Date molasses**	3.19 ^c^	1.50 ^a^	47.14 ^a^	16.03 ^b^	9.35 ^a^
**(Control, sucrose)**	(3.47 ^b^)	(0.93 ^c^)	(26.80 ^c^)	(15.07 ^c^)	(6.17 ^b^)
**LSD 0.05**	0.161	0.069	3.537	0.284	0.657

Means followed by different letters indicate significant differences between treatments according to Duncan’s test (*p* < 0.05).

**Table 3:** The correlation analysis between PHB, dry cell weight and utilized sugar.

Strains or Isolates Obtained From	Correlation Coefficient Analysis (r Value)	
	Correlation between PHB g/L and CDW (g/L)	Correlation between PHB g/L and Utilized Sugar (g/L)
**Medium (A)**	0.901	−0.012
***Bacillus paramycoides***	0.958	0.527
**Medium (B)**	0.457	−0.161
***Azotobacter salinestris***	0.987	0.051
**Medium (C)**	0.840	0.239
***Brevundimonas naejangsanensis***	0.724	0.897

Medium (A) [15]; Medium (B) [16]; Medium (C) [17].

## Data Availability

The data that supports the findings of this study are contained within the article or supplementary material and available from the corresponding author upon reasonable request.

## Supplementary Materials

The following are available online at https://www.mdpi.com/article/10.3390/polym13213801/s1: Table S1. Morphological and physiological characteristics of bacterial isolates on medium (A); Table S2. Morphological and physiological characteristics of bacterial isolates on medium (B); Table S3. Morphological and physiological characteristics of bacterial isolates on medium (C).
