# Supplementary material for: Biosynthesis of Poly-ß-Hydroxybutyrate (PHB) from Different Bacterial Strains Grown on Alternative Cheap Carbon Sources
Source: Polymers (Basel). 2021 Nov 3;13(21):3801. doi: 10.3390/polym13213801 (PMC8587160; doi:10.3390/polym13213801)
Supplement: Supplementary file 1 [file polymers-13-03801-s001.zip › polymers-1429399-supplementary.pdf]

Table S1. Morphological and physiological characteristics of bacterial isolates on medium (A)

Isolates Number	Microscopically studied					Physiological characteristics			
	Shape	Gram stain	Spore stain	Capsule stain	Motility	Indole test	Catalase test	Starch hydrolysis	Casein hydrolysis
**A1**	bacilli	+	+	+	+	+	+	+	+
**A2**	bacilli	+	+	+	+	+	+	+	+
**A3**	bacilli	+	+	-	+	+	+	+	+
**A4**	bacilli	+	+	+	+	+	+	+	+
**A5**	bacilli	+	+	+	+	+	+	+	+
**A6**	bacilli	+	+	+	+	+	+	+	+
**A7**	bacilli	+	+	+	+	+	+	+	+
**A8**	bacilli	+	+	-	+	+	+	+	+
**A9**	bacilli	+	+	+	+	+	+	+	+
**A10**	bacilli	+	+	-	+	+	+	+	+

Table S2. Morphological and physiological characteristics of bacterial isolates on medium (B)

Isolates Number	Microscopically studied					Physiological characteristics			
	Shape	Gram stain	Spore stain	Capsule stain	Motility	Indole test	Catalase test	Starch hydrolysis	Casein hydrolysis
**P1**	diplococcic	-	-	+	+	+	+	+	+
**P2**	diplococcic	-	-	+	+	+	+	+	+
**P3**	diplococcic	-	-	+	+	+	+	+	+
**P4**	diplococcic	-	-	+	-	+	+	+	+
**P5**	diplococcic	-	-	+	+	+	+	+	+
**P6**	diplococcic	-	-	+	+	+	+	+	+
**P7**	diplococcic	-	-	+	+	+	+	+	+
**P8**	diplococcic	-	-	+	-	+	+	+	+
**P9**	diplococcic	-	-	+	+	+	+	+	+
**P10**	diplococcic	-	-	+	-	+	+	+	+

Table S3. Morphological and physiological characteristics of bacterial isolates on medium (C)

Isolates Number	Microscopically studied					Physiological characteristics			
	Shape	Gram stain	Spore stain	Capsule stain	Motility	Indole test	Catalase test	Starch hydrolysis	Casein hydrolysis
**W1**	bacilli	+	+	-	+	+	+	+	+
**W2**	cocci	+	-	-	-	+	+	+	+
**W3**	short rod	-	-	+	+	+	+	+	+
**W4**	short rod	-	-	+	+	+	+	+	+
**W5**	cocci	+	-	-	-	+	+	+	+
**W6**	bacilli	+	+	-	+	+	+	+	+
**W7**	bacilli	+	+	-	+	+	+	+	+
**W8**	short rod	-	-	-	+	+	+	+	+
**W9**	cocci	+	-	-	-	+	+	+	+
**W10**	short rod	-	-	-	+	+	+	+	+
